# An Accurate Skeleton Extraction Approach From 3D Point Clouds of Maize Plants

**DOI:** 10.3389/fpls.2019.00248

**Published:** 2019-03-07

**Authors:** Sheng Wu, Weiliang Wen, Boxiang Xiao, Xinyu Guo, Jianjun Du, Chuanyu Wang, Yongjian Wang

**Affiliations:** ^1^Beijing Research Center for Information Technology in Agriculture, Beijing, China; ^2^Beijing Key Lab of Digital Plant, National Engineering Research Center for Information Technology in Agriculture, Beijing, China

**Keywords:** maize plant, 3D point cloud, Laplacian, skeleton extraction, phenotyping

## Abstract

Accurate and high-throughput determination of plant morphological traits is essential for phenotyping studies. Nowadays, there are many approaches to acquire high-quality three-dimensional (3D) point clouds of plants. However, it is difficult to estimate phenotyping parameters accurately of the whole growth stages of maize plants using these 3D point clouds. In this paper, an accurate skeleton extraction approach was proposed to bridge the gap between 3D point cloud and phenotyping traits estimation of maize plants. The algorithm first uses point cloud clustering and color difference denoising to reduce the noise of the input point clouds. Next, the Laplacian contraction algorithm is applied to shrink the points. Then the key points representing the skeleton of the plant are selected through adaptive sampling, and neighboring points are connected to form a plant skeleton composed of semantic organs. Finally, deviation skeleton points to the input point cloud are calibrated by building a step forward local coordinate along the tangent direction of the original points. The proposed approach successfully generates accurately extracted skeleton from 3D point cloud and helps to estimate phenotyping parameters with high precision of maize plants. Experimental verification of the skeleton extraction process, tested using three cultivars and different growth stages maize, demonstrates that the extracted matches the input point cloud well. Compared with 3D digitizing data-derived morphological parameters, the NRMSE of leaf length, leaf inclination angle, leaf top length, leaf azimuthal angle, leaf growth height, and plant height, estimated using the extracted plant skeleton, are 5.27, 8.37, 5.12, 4.42, 1.53, and 0.83%, respectively, which could meet the needs of phenotyping analysis. The time required to process a single maize plant is below 100 s. The proposed approach may play an important role in further maize research and applications, such as genotype-to-phenotype study, geometric reconstruction, functional structural maize modeling, and dynamic growth animation.

## Introduction

Plant phenomics has gained more attention as a promising intervention in recent years, because it still remains a bottleneck that limits genetic gain in breeding programs (Araus et al., 2018). Accurate and high throughput measurement of high-dimensional plant morphology across plant development is the essential component of plant phenotyping (Houle et al., 2010). Traditional phenotyping technologies are usually time consuming, low throughput, and labor intensive, which is far behind the development of genomics, although efforts have been made to improve phenotyping efficiency (Yang et al., 2013; Zhang et al., 2017). Substantial changes and improvements in phenotyping technologies for crops will be required for a long term (Tester and Langridge, 2010).

Due to the complexity of plant morphometrics, many kinds of sensors need to be involved to acquire the morphological data of plants, including RGB cameras (Chaivivatrakul et al., 2014), depth cameras (Hu et al., 2018), LiDAR (Jimenez-Berni et al., 2018), and multispectral sensors (Potgieter et al., 2017), and thousands of images and point clouds are generated. Thus automatic data processing algorithms and software play an important role to covert these huge amounts of raw data into clear meaning phenotyping traits, and further into knowledge (Tardieu et al., 2017). However, there is none uniform solution for all kinds of plants, because plant morphology are diverse for different species. Therefore, specialized plant phenotyping algorithms have to be developed for architecture determined plants.

Maize is one of the most widely grown crops worldwide and the structure is relatively simple with one stem and several leaves. Its levels of phenotypic and genetic variation derived lots of attention for phenotyping algorithm development as a model plant (Yan et al., 2011). Recent developments in unmanned aerial vehicle (UAV) have provided research opportunities in assessing plot and field scale phenotyping traits (Zaman-Allah et al., 2015; Yang et al., 2017). Though canopy scale traits, including normalized difference vegetation index (NDVI), leaf area index (LAI), nitrogen stress, and plant height, could be derived using multispectral images and LiDAR, it is impossible to derive more detailed phenotyping traits, due to the space resolution of the raw data and occlusion of nearby plants (Pfeiffer et al., 2018). To meet the needs of ideal plant type genotype breeding (Zhang et al., 2017), more detailed traits at plant or organ scale such as leaf length, leaf area, and leaf curvature have to be obtained. Thus, high throughput phenotyping platforms (Yang et al., 2014; Cabrera-Bosquet et al., 2016; Guo et al., 2016) in controlled environment were set up to acquire the morphological data of individual plants. Consequently, automatic data processing algorithms have to be developed to deal with these raw data.

Most of the morphological data of individual plants exist in terms of two-dimensional (2D) images or three-dimensional (3D) point clouds (Vazquez-Arellano et al., 2016). The first process is to segment these data into independent organs with semantics. Then ideal methods are to 3D reconstruct each organ into fine organ meshes (Pound et al., 2014; Yin et al., 2016; Gibbs et al., 2017; Thapa et al., 2018) and then all the morphological phenotyping traits could be derived, such as leaf length, leaf width, and accurate leaf area. However, it is hard to obtain complete morphology of an individual plant for most cases. Therefore, an alternative way to parse these organ traits is curve skeleton extraction from 3D point clouds (Cornea et al., 2007).

Skeleton extraction from 2D images is relatively easier using image thinning algorithms. However, phenotyping traits, such as leaf length and leaf azimuth, have to be calibrated owing to one-dimensional data missing (Klukas et al., 2014; Brichet et al., 2017; Zhang et al., 2017). It is much more sophisticated of 3D skeleton extraction of branched structure plants, because another information has to be considered to determine the skeleton curve. The most notable curve skeleton extraction algorithms are rotational symmetry axis (ROSA)-based (Tagliasacchi et al., 2009) and L_1_-medial skeleton of point clouds (Huang et al., 2013) for most incomplete point clouds. These methods are feasible for cylindrical structure point clouds while not suitable for narrow and long plant leaves, because there are deviations between extracted curve and leaf veins. The point cloud of trees without leaves are featured as cylindrical branched structure, which are suitable for skeleton extraction (Livny et al., 2010; Delagrange et al., 2014; Wang et al., 2014). Laplacian skeleton extraction (Au et al., 2008) from point clouds is another widely used method, which has been successfully used in performing skeleton extraction without the need for mesh reconstruction (Su et al., 2011). Extracted curves from point clouds of long and narrow leaves using these methods directly are unsatisfactory, and could not meet the needs of accurate phenotyping traits analysis (Zhang et al., 2017). Meanwhile, there is an increasing maize plant morphological data acquisition equipment (Chaudhury et al., 2017; Gibbs et al., 2018), methods (Hui et al., 2018), and platforms (Cabrera-Bosquet et al., 2016), obtaining huge amount of high-quality point clouds. Therefore, developing automatic skeleton extraction from point clouds for maize leaves and plants is urgent for phenomic study.

In this study, we applied and improved Laplacian skeleton extraction method on point clouds of maize plants to achieve the following three goals: (1) extract the skeleton curve of maize leaves with less deviation of the stem and veins; (2) improve the accuracy of phenotyping traits of maize plants, such as leaf length and leaf azimuth, which needs to be calibrated in image based methods; and (3) the proposed method is able to solve the point clouds of the all growth periods of maize plants.

## Materials

**FIGURE 1:**
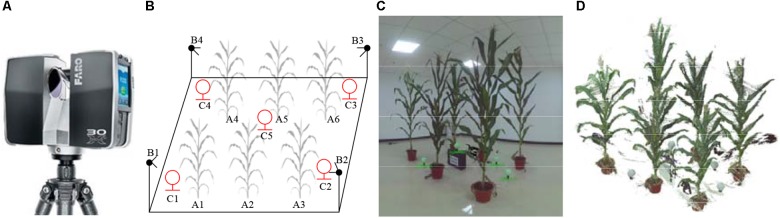
Maize plant point cloud scanning scene and point cloud visualization. **(A)** Faro Focus^3D^ X130 3D scanner. **(B)** 3D scanning arrangement: A1∼A6 are maize plants, B1∼B4 are the positions where the scanner placed, and C1∼C5 are the scanning positioning balls used for software registration placed in different heights. Adjacent plants are generally arranged with the distance of 0.8–1 m, and the scanner positions were 1 m away from the nearest plant. **(C)** Photograph of scanning plant arrangement. **(D)** Raw 3D point cloud visualization corresponding to **(C)**. White lines were observed in **(C,D)**.

The experiment was conducted in the farm of Beijing Academy of Agricultural and Forestry Sciences (39.94 N, 116.28 E) and maize plants were planted in the field with the density of 6 plants/m^2^ (the row distance is 60 cm). Three cultivar plants, including ZhengDan958 (ZD958), JingKe968 (JK968), and XiangYu335 (XY335), were selected randomly from jointing to silking stages. Two sample plants of each three cultivars were selected. The plants were excavated with 20 cm roots and soil into pots and moved indoor without any morphological damage to avoid generating noise by wind and other external factors. Many approaches are available for 3D point cloud data acquisition of maize plants, including multi-view stereo (MVS) 3D reconstruction, 3D scanning, and 3D synthesis by 2D LiDAR. A terrestrial laser scanner Faro Focus^3D^ X130 was used here to acquire the point clouds of selected plants indoor. The scan resolution was 0.035° horizontally and vertically with a maximum distance of 130 m (Johnson and Liscio, 2015; Seidel et al., 2015). There is integrated camera of the scanner, so the color information was also acquired simultaneously with the point clouds. To increase the scanning efficiency, six plants were placed in two rows and three plants per row and were scanned together each time, as shown in Figure 1. To promise the point cloud completeness of each plant, four scanning stations were arranged sequentially around the target area (Figure 1B). Four stacks of scanned data at the mentioned position above of different azimuths were obtained. We registered the four stacks under the same coordinate system and merged them into a complete group of point cloud using the supporting software SCENE provided by FARO. Outlier points, including room walls and floors, were removed also by SCENE. Each station scanning takes 5 min. The point cloud contains 3D coordinate and color information of all the vertices.

## Methods

**FIGURE 2:**
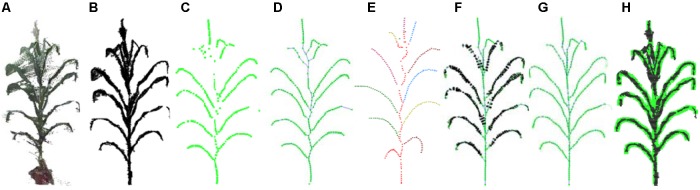
Workflow of skeleton extraction from point clouds of maize plants. **(A)** Input point cloud. **(B)** Point cloud after denoising. **(C)** Key points contraction of the skeleton using Laplacian. **(D)** Skeleton point resampling and connection. **(E)** Stem and leaf point recognition. **(F)** Skeleton points calibration. **(G)** Final skeleton extraction result. **(H)** Matching visualization of the input point cloud and the extracted skeleton.

The pipeline presented here involved five steps, namely, (1) point cloud denoising; (2) point cloud contraction using Laplacian; (3) adaptive sampling, (4) skeleton point connection, and (5) skeleton curve correction. The overall workflow is described in Figure 2.

### Point Cloud Denoising

In the 3D scanning process, even slight wind like human moving around the plants may lead to overlaps and offsets in the resultant point clouds. In addition, the point cloud of pots, used to load and hold the maize plant, also needs to be detected and deleted. Therefore, we have to pre-process the point cloud before further operation. The obtained point clouds were dense and the number of points describing a grown up maize plant could reach up to 50 thousand points. In order to improve the efficiency of the following algorithm, a uniform simplification algorithm (Han et al., 2015) was applied to reduce the density of the dense point clouds. Empirical results showed that when the density of the point cloud of each maize plant was reduced to about 10 thousand points, the accuracy of the algorithm would not be affected too much and also be satisfactory.

#### Pot Points Detection

**FIGURE 3:**
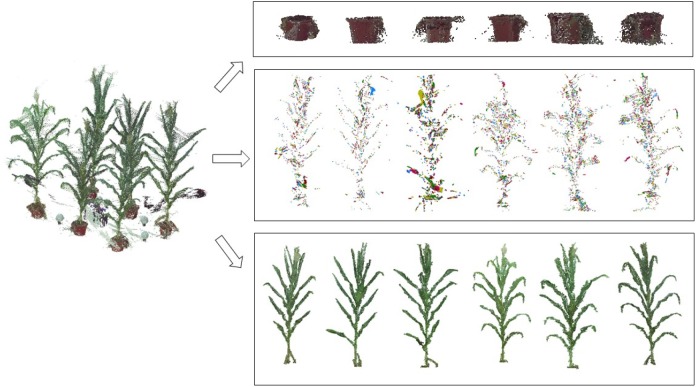
Illustration of the denoising process; the 3D scanned data are separated into three parts: pot noise, plant noise, and point cloud of individual plants.

The pots were usually used in many maize plant phenotyping platforms, such as potting plants in greenhouse and transplanting in field cases, they play an important role in holding the plant stable. To detect and delete the points of the pots, a color difference denoising method (Dun et al., 2011) was used to filter the noise of the pots. First, the RGB color values of the pot, manually picked originally under stable light conditions of data acquisition site, are sampled to form a noise color list. Then, the color difference *D* between each point of the noise color list point and the point cloud P is calculated using the equation set (1) and (2). If the color difference value *D* is less than a threshold (here it is 0.1), it indicates that the point is a noise point and is thus removed.

(1) $$D(p_{i},p_{j})=2\left|\frac{\max(\eta_{i},\eta_{j})}{\min(\eta_{i},\eta_{j})}-1\right|\theta$$

(2) $$\theta =\arccos\;\left(\frac{\vec{p_{i}}\vec{p_{j}}}{\left|\vec{p_{i}}\right|\left|\vec{p_{j}}\right|}\right)\frac{255}{\pi /2}$$

where *p_i_(r_i_,g_i_,b_i_)* and *p_j_(r_j_,g_j_,b_j_)* are two points, *r, g*, and *b* are the color components of each point, with $\vec{p_{i}}$ and $\vec{p_{j}}$ as the corresponding vectors, and η = (*r* + *g* + *b*)/3, and $\vec{p}$ = (*r* -μ, *g* -μ, *b*-μ) Figure 3 illustrates the point clouds of detected pots.

#### Plant Denoising

**FIGURE 4:**
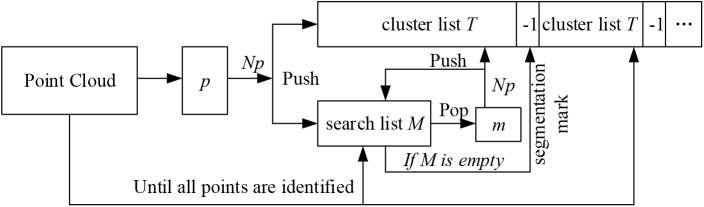
Flowchart of the clustering algorithm.

A near propagation clustering algorithm was proposed to filter the scanning noise of plants, inspired by the *K* nearest neighbor clustering algorithm (Connor and Kumar, 2010). There are five steps of this algorithm (Figure 4).

(1) Randomly select any point *p* in the point cloud, and identify the nearest *N_P_* neighboring points within a distance parameter *r* around the point *p*. Then mark the point *p* and its *N_p_* neighbors as the access state. Here *r* = 0.012*l*, where *l* is the longest diagonal of the point cloud bounding box.
(2) Store the point *p* and its *N_p_* neighbors into a cluster list *T*, and store the *N_p_* neighbors into a search list *M*.
(3) Traverse through the list *M*. For each point *m* in *M*, find its nearest neighboring points also within the range of *r*. Then for each point *p_m_* in *m*’s neighborhood, if the point *p_m_* has not been visited, save it to the cluster list *T* as well as the search list *M.* Once visited, delete it from the search list *M*. The process continues till the linked list *M* is empty and an empty index (the index is -1 in cluster list T in Figure 4) is inserted to segment the cluster list *T*.
(4) The nearest neighbor clustering operations described above are iteratively applied until the states of all the points in the cloud are marked.
(5) The clustering point cloud is sorted according to the number of points in the cloud, and the clusters with number of points less than the threshold are considered as the noisy point clouds and are deleted. In practice, the threshold is calculated by: $n=0.5\sum_{i=0}^{num}d\mathrm{en}sity(p_{i},\;r)/num$, where *density*(*p, r*) is the density of the point cloud (Wu et al., 2017). In order to improve the efficiency, the parameter *num* was set 10, determined through many numerical experiments, and these *num* points are randomly selected in the point cloud. Figure 3 shows the point clouds after plant denoising and the corresponding noises.

### Laplacian Point Cloud Contraction

The point cloud of a maize plant is iteratively contracted using the classical restricted Laplace operator (Cao et al., 2010). It is represented as follows:

(3) $$\left[\begin{array}{l} W_{L}^{t}L^{t}\\ W_{H}^{t} \end{array}\right]P^{t+1}=\left[\begin{array}{l} 0\\ W_{H}^{t}P^{t} \end{array}\right]$$

Here *P* is the processing point cloud. *L* is the weighted cotangent matrix which is constructed using the Delaunay neighborhood points; *W_L_* and *W_H_* are the diagonal matrices, where *W_L_* controls the intensity of contraction, and *W_H_* controls the intensity of the original position. This ensures that the point cloud of the contraction equation moves along the estimated normal direction.

**FIGURE 5:**
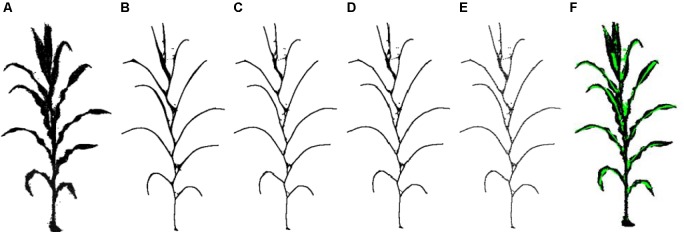
Schematic diagram of contraction iteration process. **(A)** Point cloud after denoising also as the input of the Laplacian contraction. **(B–E)** One to four times iterations. **(F)** Contraction result matching the point cloud after denoising. Note: the green points are skeleton points and black dots represent the point cloud. The same expressions are used in the rest of this paper.

The iterative shrinkage process can be described as follows: Eq. 3 is used to solve and *P^t+1^* is derived. Then *W_L_* and *W_H_* are updated using Eq. 4. Here $S_{i}^{t}$ and $S_{i}^{0}$ are the current and the initial neighborhood length of the contraction point *p_i_*, respectively. This allows us to obtain the new point cloud *P^t+1^*. Next, reconstruct the Laplacian matrix *L^t+1^* using the new point cloud *P^t+1^*.The termination condition of the iteration is $W_{L}^{t+1}/W_{L}^{t}$ < 0.01 or the count of iteration is greater than 20. Generally, after a finite number of iterations, the point cloud *P* shrinks to the shape of the skeleton. As shown in Figure 5, the maize plant point cloud has shrunk to a better skeleton shape after four iterations of contraction.

(4) $$W_{L}^{t+1}=S_{L}W_{L}^{t},W_{H,i}^{t+1}=W_{H,i}^{0}S_{i}^{0}/S_{i}^{t}$$

### Adaptive Sampling

Point clouds of maize plants shrunk into skeleton shape using the above Laplacian shrinkage. However, the number of points remains the same even though the shape changes. It is necessary to adaptively sample and identify the key points of the skeleton in the point cloud. In order to maintain the geometric characteristics of the branches, different spherical radius sampling technology was used at the intersections and the branches (leaves and stems). Radius of the sampling sphere is smaller at the intersections than in the branches. To determine whether a point *v* is located at the intersections, the directionality degree *l*(*v*) of point *v* is introduced to describe the linear trend at the current point (Su et al., 2011). This directionality degree *l*(*v*) is obtained using the 3 × 3 covariance matrix *C* of the sampled sphere (Eq. 5) as expressed as follows:

(5) $$C={\left[\begin{array}{c} v_{1}-\overset{-}{v}\\ ...\\ v_{k}-\overset{-}{v} \end{array}\right]}^{T}\left[\begin{array}{c} v_{1}-\overset{-}{v}\\ ...\\ v_{k}-\overset{-}{v} \end{array}\right]$$

(6) $$C\cdot V_{l}=\lambda_{l}\cdot V_{l},l\in \{0,1,2\}$$

(7) $$l(v)=\frac{\lambda_{2}}{\lambda_{0}+\lambda_{1}+\lambda_{2}}$$

where v_1_,...v_k_ are local *k*-neighborhood of *p*; $\bar{v}$ is the centroid of the neighbors of *p*, namely, $\bar{v}=\frac{1}{k}\sum_{i\in k}v_{i}$. *V_l_* is the eigenvector of matrix *C*, and is the eigenvalue of matrix *C.* It λ_*l*_ is assumed that λ_0_ ≤ λ_1_ ≤ λ_2_. The closer *l*(*v*) is to 1, the smaller λ_0_ and λ_1_ are compared to λ_2_; and hence, the more points around *v* are aligned along a branch.

**FIGURE 6:**
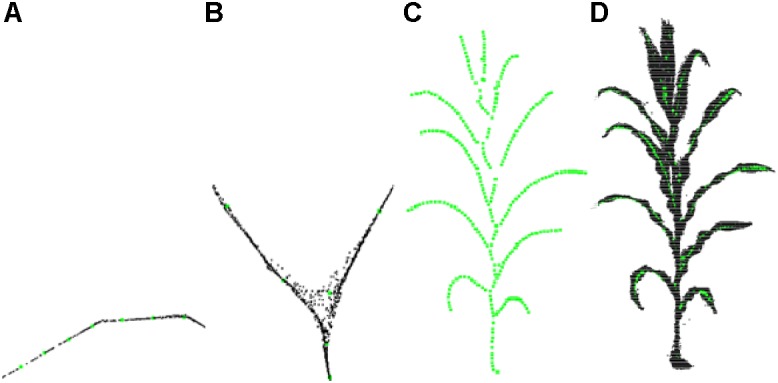
Adaptive sampling of skeleton points. **(A)** Leaf points sampling. **(B)** Branching point sampling. **(C)** Sampling result. **(D)** Matching effect of sampling point and original point cloud.

Experiments show that *l*(*v*) is greater than 0.9 at the intersections and whereas otherwise. After the adaptive sampling processing, the shrinkage point cloud is reduced to the skeleton key points, and the resultant skeleton has a sparse number of points. As shown in Figure 6, the sampling accuracy obtained is better after the adaptive sampling results and the obtained results match the original point cloud.

### Skeleton Point Connection

**FIGURE 7:**
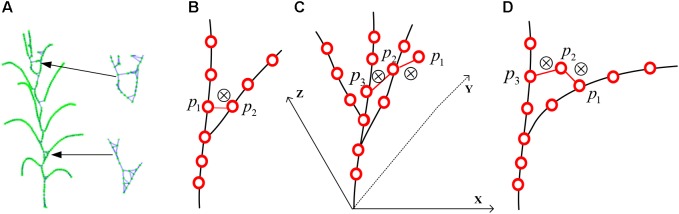
Schematic diagram of skeleton connection and closed loop breaking, “⊗” represents disconnection. **(A)** Three nearest neighbor skeleton connection. **(B)** Triangle closed loop. **(C)** Approximately coplanar. **(D)** Growth direction convergence.

**FIGURE 8:**
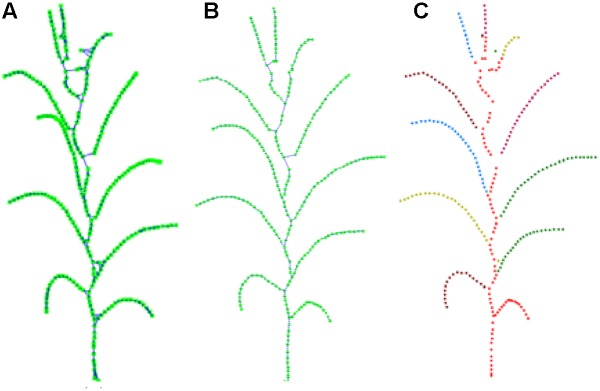
Key point connection and extraction result of stem and leaf. **(A)** K (*k* = 3) nearest neighbor connection. **(B)** Closed loop removal. **(C)** Segmentation and recognition of stem and leaves in different colors.

To estimate the phenotyping traits of maize plant using the extracted skeleton curve, semantic connection key points have to be determined. Every key point in the extracted skeleton has a maximum of three neighboring points since maize plant has a single branch structure. Therefore, if three nearest neighboring points are connected, the skeleton will end up as an undirected digraph. However, as shown in Figures 7A, 8A, two types of closed connection loop errors might appear: (1) triangle closed connection loop (Figure 7B) or (2) polygon closed connection loop. The skeleton of the maize plant has the characteristics that nearby points are approximately coplanar and show convergence in the direction of the growth. Therefore, we construct the constraint model of the morphological structure of the maize plant and apply the connected weight equation to break the closed loops:

(8) $$W=[W_{s}/\max(W_{si})]+[W_{c}/\max(W_{ci})]$$

(9) ax+by+cz+d=0

(10) $$e=\sum_{i=1}^{n}d_{i}^{2}\to \min$$

(11) $$W_{s}=d_{i}+d_{i+1}$$

(12) $$W_{c}=\alpha_{i}+\alpha_{i+1}$$

Here *W_s_* and *W_c_* are the approximate coplanar weight and the growth weight of the edge, respectively. For the approximate coplanar weight (shown in Figure 7C), the least squares algorithm (Zeng et al., 2012) is used to fit the closed loop nodes. And the minimum distance from each point to the plane (Eq. 10) is used as the constraint condition for solving for the fitting plane, and *a, b, c*, and *d* are the parameters of the plane equation (Eq. 9). The coplanar weights of the edges are the sum of the distances from the two fixed points to the fitted planes (Eq. 11). To determine the growth weights (shown in Figure 7D), the angle between the edge and its two adjacent edges in the direction of the growth is calculated (Eq. 12). In order to ensure the equality while comparing the weights, we rescale and normalize the weights (Eq. 8). Subsequently, to obtain the weight of each connecting edge, we disconnect the edge from the edge with the maximum weight (shown in Figure 7D).

After the closed loop searching and processing as explained above, the skeleton of a maize plant connected as a tree structure is obtained, as shown in Figure 8B. And the plant skeleton is easily segmented into leaves and stem skeleton nodes by the three connected points in the skeleton. The segmented result is visualized as Figure 8C.

### Skeleton Point Calibration

**FIGURE 9:**
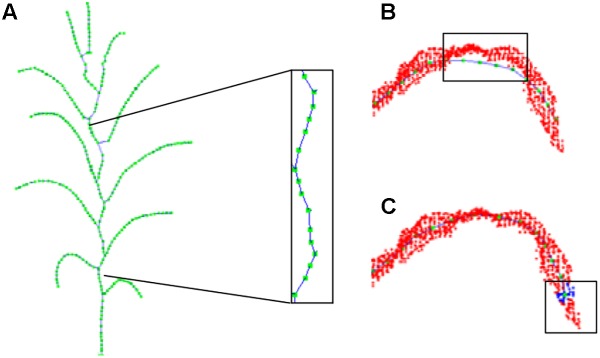
Offset illustration of contracted skeleton point. **(A)** Stem skeleton curving. **(B)** Vein points deviation. **(C)** Tip nodes short missing.

The extracted skeleton of a maize plant using the above procedure contains many curving sections, especially the sections near the stem, where the skeletons ought to be linear straight. This severely affects the further estimation of phenotyping traits. Existing problems of the skeleton (Tagliasacchi et al., 2009; Cao et al., 2010) that must be corrected here could be summarized as two categories: (1) stem curving, as shown in Figure 9A, (2) too much deviation from the vein (as shown in Figure 9B), and tip missing at the end of the leaves (as shown in Figure 9C). Therefore, resampling strategy is adopted to calibrate the skeletons.

#### Stem Skeleton Calibration

**FIGURE 10:**
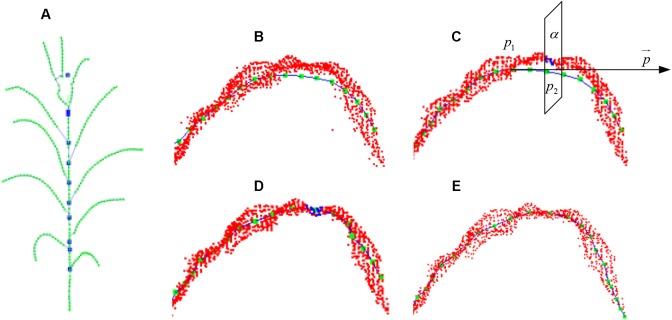
Skeleton calibration process. **(A)** Calibrated stem skeleton. **(B)** Vein curve error. **(C)** Cutting plane perpendicular to the tangent direction of the vein curve. **(D)** Cutting points of vein curve. **(E)** Calibrated vein curve.

The bent points of extracted stem skeleton are generated because the attraction of the adjacent leaves while executing the Laplacian contraction algorithm. However, this only affects the nearby points, and the stem nodes which are away from the leaves are not affected. Therefore, the stem skeleton is calibrated using the unaffected points of the stems. According to the segmentation and recognition of stem and leaves in Section “Stem Skeleton Calibration,” leaf growth point is defined as the interaction points of leaf with adjacent stem. The leaf growth points segment the entire stem into several sections. For each stem section, the extracted stem points in previous processes, barring the leaf growth points at both ends of the considered stem section, are used to fit a straight line segment based on least square distance. Finally, all the extracted skeleton points on the current stem section are projected on the new fitted line segment and are thus calibrated, as shown in Figure 10A.

#### Leaf Skeleton Calibration

To calibrate the skeleton points along the vein deviation (shown in Figure 10B), especially for the curved arc that usually located between the highest position on the blade and its base, we sample and calibrate all the deviated points of the skeleton starting from the blade base toward the tangential direction. First, the tangent line $\vec{p}$ = *p*_1_*p*_2_ is constructed through the adjacent vein skeleton points *p*_1_ and *p*_2_. Then a cutting plane α (Figure 10C) perpendicular to $\vec{p}$ through *p*_2_ interacts with the original point cloud of the leaf as the intersection set (Figure 10D). The central point of the set is the calibrated point of *p*_2_ and located on/near the vein curve. The excessive contraction problem of leaf tip is solved in the same way. Whereas the cutting plane here is constructed at a certain length interval, and the most suitable point on this plane is identified as the leaf tip. As shown in Figure 10E, we continue to move forward of the cutting plane until the entire cutting point cloud is empty, and the vein skeleton is calibrated.

## Results

The algorithm was implemented using OpenGL graphics library and Point Cloud Library (PCL; Rusu and Cousins, 2011) in VC++ 2010 development platform. The algorithm was integrated into the software PlantCAD_Maize, which is one of our serious plant modeling software PlantCAD (Lu et al., 2014). The algorithm runs on a desktop workstation with the configuration of core i5 processor and 4 GB memory.

### Visualization Results

**FIGURE 11:**
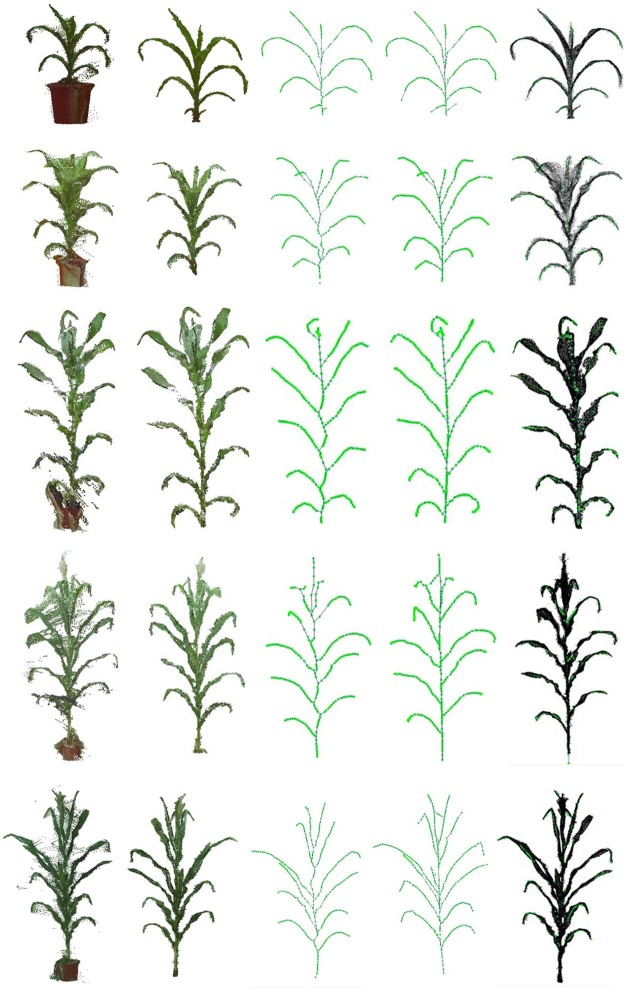
Skeleton extraction procedure visualization of different cultivars and growth stages. From top to down, we have ZD958 of jointing stage, ZD958 of flare opening stage, JK968 of silking stage, XY335 of silking stage, and ZD958 of silking stage. **(A)** Input point cloud. **(B)** Plant point cloud after denoising. **(C)** Extracted skeleton using Laplacian and key point connection. **(D)** Final extracted skeleton after calibration. **(E)** Matching result of extracted skeleton and input point cloud without pot.

**FIGURE 12:**
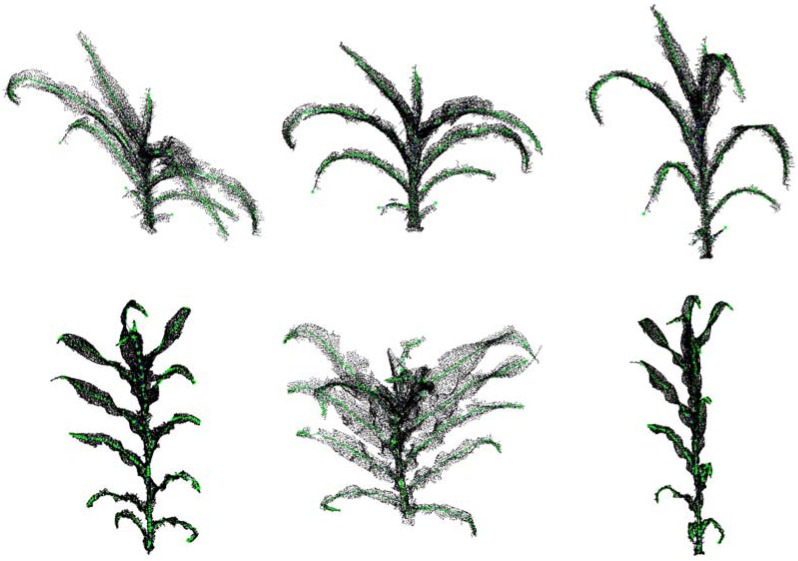
Matching visualization of extracted skeletons and original point clouds in different angle of views. The top row is ZD958 of jointing stage (corresponding to the first row in Figure 11), and the bottom row is JK968 of silking stage (corresponding to the third row in Figure 11).

Maize plants of different cultivars and growth stages were selected to evaluate the performance of the algorithm. Figure 11 shows five visualization results processed by the approach, including three growth stage plus three different cultivars of maize plants. Figure 12 shows two corresponding matching visualization in different angle of views. The matching results of extracted skeleton and the original point cloud demonstrate that the proposed algorithm has a good performance and adaptability, and the approach is feasible for different plant size and different plant type plants.

### Accuracy Analysis of Phenotypic TraitsUsing the Extracted Skeleton

**FIGURE 13:**
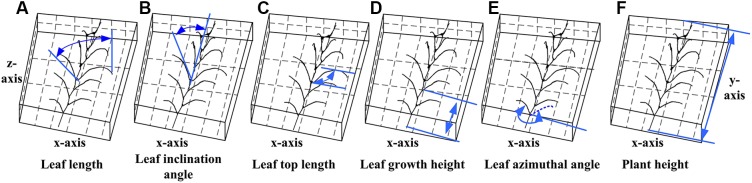
Measurement principle of phenotypic parameters. **(A)** Leaf length: the sum of the distance between the key points of the skeleton from the base point to the tip of the leaf. **(B)** Leaf inclination angle: the angle between the tangent of the leaf base and the axis of the stem. **(C)** Leaf top length: the length from the base of the leaf to the highest point of the leaf. **(D)** Leaf growth height: the vertical distance from the base of the leaf to the base of the stem. **(E)** Leaf azimuthal angle: the angle between the leaf and the base leaf (counter clockwise). **(F)** Plant height: the vertical distance from the highest point of the plant skeleton to the base point of the plant.

The extracted skeleton of maize plant contains segmentation of organs and semantic meaning of each organ, which allows rapid estimation of phenotyping parameters, including plant height and volume in plant scale, and leaf length, leaf inclination angle, leaf azimuth, and more traits in organ scale. Detailed morphological parameters are explained in Figure 13 (Zhang et al., 2017). Particularly, the leaf azimuthal angles are calculated through comparing the difference between the target leaf and the lowest leaf, where the azimuth of the lowest leaf is assumed to be 0.

**FIGURE 14:**
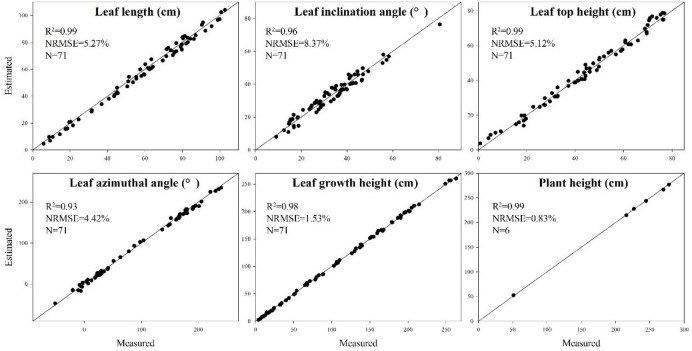
Comparison of six phenotyping traits derived using the extracted skeleton from point clouds and measured 3D digitizing data of six same maize plants. There are 71 leaves in total of the six plants.

To verify the accuracy of the skeleton extraction algorithm, 3D digitization data of corresponding maize plants were acquired using a 3D digitizer (Wen et al., 2017). The equipment used here is FastRak (Polhemuns, United States; Everhart et al., 2009). The acquired data do not need any further processing due to the semantic characteristics of each obtained point (Sinoquet et al., 1998). Therefore, the skeleton data obtained using the 3D digitizer could be considered as the real skeleton if we ignore the influence of any human error. So we used these 3D digitizing data as the ground truth for evaluating the algorithm. Figure 14 shows six estimated phenotyping parameter (leaf length, leaf inclination angle, leaf top length, leaf azimuthal angle, height of leaf position, and plant height) comparison between the extracted skeleton from point cloud and 3D digitizing data of the same six plants used in our experiment. The average normalized root-mean-square error (NRMSE) of plant height is the smallest, which is 0.83%, and the NRMSE of leaf inclination angle is the largest, which is 8.37%. The *R*^2^ of all phenotypic parameters are above 0.93, indicating that there is a high consistency of estimated phenotyping traits between the extracted skeleton and the measured data. In particular, the plant height can be derived by calculating directly from the point cloud for most of the plants. However, some of the plants were declining and the plant height calculation was not so accurate for these plants. Thus, plant skeletonization steps, containing the calibration of stem orientations, would promise a more satisfactory result of plant height estimation.

We also compare the proposed algorithm with the previous constrained Laplacian smoothing (CLS) point cloud contraction algorithm (Su et al., 2011). Plants of three cultivars were segmented into up and down layers by the ear-leaf, and five phenotyping parameters were estimated of the two layers. Table 1 gives the average of the five phenotyping parameters estimated using these two methods. It shows that calculation error derived using the proposed approach is almost half of the CLS algorithm, and the errors are relatively lower for different cultivars of maize plant phenotyping traits.

**Table 1:** NRMSE (%) comparison of five phenotyping traits derived using the extracted skeleton between our and CLS method.

Cultivar	Layer	Leaf length		Leaf inclination angle		Leaf top length		Leaf azimuthal angle		Height of leaf position	
						
		Our	CLS	Our	CLS	Our	CLS	Our	CLS	Our	CLS
JK968	Up	5.28	11.20	6.70	13.00	6.64	13.64	5.36	8.60	1.03	1.47
	Down	4.14	7.09	8.85	16.94	5.28	8.16	3.27	6.25	2.96	4.95
ZD958	Up	5.84	10.45	12.19	25.78	5.13	7.26	2.91	4.90	1.79	4.94
	Down	5.28	7.92	7.19	12.78	6.15	12.49	5.44	9.07	4.43	7.73
XY335	Up	5.12	10.71	12.64	23.21	5.68	14.22	5.07	7.84	0.85	1.75
	Down	3.12	5.15	8.10	12.72	4.53	6.79	4.69	7.40	1.73	2.94
Average		5.27	9.89	8.37	15.83	5.12	9.60	4.42	6.91	1.53	2.77


### Efficiency

**FIGURE 15:**
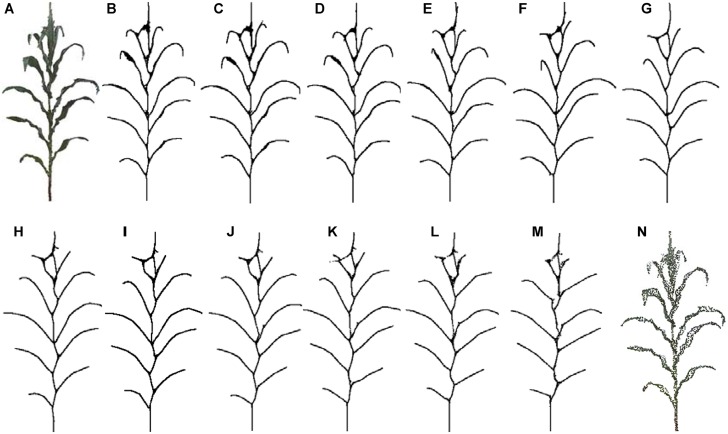
Skeleton contraction effect of different number of input points, **(A)** and **(N)** are point clouds of maize plants with points 142,579 and 2,615, respectively. **(B)** Num = 142,579. **(C)** Num = 100,213. **(D)** Num = 68,789. **(E)** Num = 39,857. **(F)** Num = 25,110. **(G)** Num = 15,820. **(H)** Num = 11,047. **(I)** Num = 8725. **(J)** Num = 6805. **(K)** Num = 5044. **(L)** Num = 3826. **(M)** Num = 2615.

Depending on the high accuracy of the 3D scanner used, high density point clouds of maize plants are obtained. Taking a silking stage maize plant as example, its point cloud contains 140 thousand points, as shown in Figure 15. According to the proposed approach, the skeleton could be extracted with different sampling point number as the input. In Figure 15B–M, it could be observed that the shape and structure of the skeleton still keeps a good approximate effect when the number of sample points is kept at about 10 thousand points. Therefore, we select 10 thousand points as the optimal sampling number for a maize plant of the approach.

**FIGURE 16:**
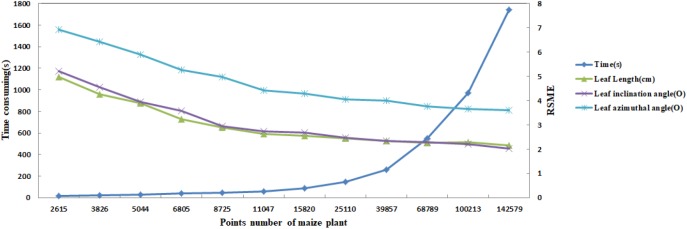
Comparison of calculation efficiency and RMSE dynamic of three phenotyping traits for different sampling point number of maize plants.

In practice, the longest time consumed by the algorithm is *K* nearest neighborhood calculation of the input point cloud. Therefore, the computational efficiency of the algorithm can be improved reducing the number of point clouds as much as possible. However, as mentioned above, the optimal input point cloud number is 10 thousand. The total time taken for the extraction of the skeleton of a maize plant with about 10 thousand points is about 60 s excluding device scanning time. Figure 16 illustrates the time consumed of the algorithm increases with the point cloud size increases, while the RMSE of leaf length, leaf inclination angle, and leaf azimuth angle becomes smaller. And the approach of this paper does not significantly increase the time consumption compared to the CLS algorithm (Su et al., 2011). The calculation will become faster if the approach runs on high configuration computers. This is much faster than traditional manual skeleton data acquisition using 3D digitizers.

## Discussion

The processing approach reported here can accurately extract skeleton from 3D point cloud of maize plant, it can approximate the geometric centric of plant organs, such as the vein of the leaves and the central axis of the stem. On the basis of which, phenotyping traits could be calculated accurately, which provides useful information for genomic studies and/or breeding programs (Fiorani and Schurr, 2013; Fahlgren et al., 2015; Yang et al., 2015). At present, most phenotyping platforms using image-based skeleton extraction algorithms (Cabrera-Bosquet et al., 2016; Zhang et al., 2017) for calculating phenotyping traits, which is difficult to extract morphological parameters in the other dimensional, such as leaf width and leaf azimuthal angle. The proposed approach provides an effective solution of the above issue using 3D point cloud data as its input. At present, there are many routines to obtain high accuracy and high-quality point clouds of plants, including data acquisition using 3D scanners (Yin et al., 2016), MVS-based 3D reconstruction (Gibbs et al., 2018), 3D synthesis by 2D LiDAR combined with plant rotation (Thapa et al., 2018). Using these 3D point clouds of plants, this algorithm will play an important role in further maize phenotyping data processing and analysis (Zhang et al., 2017).

Besides estimating phenotyping parameters for genotype-to-phenotype study, plant skeleton also plays an important role in many virtual agricultural applications, such as geometric modeling, animation, shape deformation, and growth simulation (Wade and Parent, 2002; Yan et al., 2008). Combined with delicate leaf meshes which present detailed morphological differences of diverse maize cultivars (Wen et al., 2017), it is possible to design and reconstruct various 3D maize plant models using the extracted skeleton, using skeleton driving mesh deformation technologies (Yan et al., 2008). High-quality reconstructed plants and canopy may promote the development of functional structural plant models (FSPMs; Vos et al., 2010) to a more accurate level. For example, accurate reconstructed 3D canopy model provides a precise basis for light distribution simulation and verification (Chelle and Andrieu, 1998; Cieslak et al., 2008), as well as further photosynthesis modeling (Evers et al., 2010).

We also note three limitations of the proposed approach. First, due to the 3D resolution of point cloud differs to most of the plant organs, the extracted skeleton of tassel and female ears are not satisfactory. Some of the tassels are extracted as only one truck, missing the other branches. And the female ears are always integrated into the ear leaf skeleton. This may be resolved when the point cloud density and accuracy of tassel and female ear improve. Second, the upper leaves of the maize plant are usually compact or incompletely unfolded, which makes it difficult to completely separate the leaf point cloud from the stem, and the compact structure shows that the leaves and stalks block each other, resulting in a loss of points, which brings higher error rate of upper than lower leaves (see Table 1). At present, some of the 3D maize phenotyping approaches successfully solved the 3D skeleton extraction and reconstruction of early growth stage maize plants, but plants after silking stage remain a problem due to the occlusion of upper parts of the plants (Garrido et al., 2015; Thapa et al., 2018). In practical applications of our approach, these deviations can be corrected through simple human interaction, and we provide interactively modified functional modules in the algorithm integrated software PlantCAD (Lu et al., 2014). At last, this approach is not applicable for outdoor and *in situ* data acquisition when the wind speed is greater than 2 m/s. The presence of wind would create noises which seriously affect the quality of the obtained data decreasing the accuracy of the extracted skeleton, especially at the intersection of the blade and the stem. In the event of no winds and stable radiations in field conditions, the target plants must be sparse enough for scanning to avoid intersection and occlusions of adjacent plants, which would decrease the noise and deficiency of points. In this case, the 3D point cloud acquired would be satisfactory for this approach and same skeleton would be extracted as indoors. Therefore, our future work will improve this approach that could be more robust and effective for rough point clouds of maize plants.

## Conclusion

This paper presents a skeleton extraction approach of a maize plants from 3D point clouds. Five process including point cloud denoising, point cloud contraction using Laplacian, adaptive sampling, skeleton point connection, and skeleton curve correction are sequentially applied on input point cloud of maize. Consequently, a semantic and accurate maize plant skeleton will be derived. Phenotyping parameters of plant and organ scale could be calculated on the basis of the extracted skeleton. Experiments performed using the proposed approach on different cultivars and growth stages of maize plants show that the extracted skeleton was highly consistent with the original point cloud. The complete processing of the algorithm takes less than 100 s of a maize plant. The process is therefore robust, accurate, and efficient. Therefore, this algorithm can provide technical support for the development of automatic tools for maize phenotyping data processing and analysis.

## Author Contributions

SW proposed and developed the approach, and wrote methods of this article. WW wrote other parts of the article and acquired the 3D point clouds of plants. BX evaluated the accuracy of the approach. XG designed the study. JD and CW improved the approach in some details. YW acquired the 3D digitizing data.

## Conflict of Interest Statement

The authors declare that the research was conducted in the absence of any commercial or financial relationships that could be construed as a potential conflict of interest.
